# A Low-Cost Data Acquisition System for Automobile Dynamics Applications

**DOI:** 10.3390/s18020366

**Published:** 2018-01-27

**Authors:** Alejandro González, José Luis Olazagoitia, Jordi Vinolas

**Affiliations:** Industrial Engineering Department, Universidad Antonio de Nebrija, 55 Pirineos Street, 28040 Madrid, Spain; agonzalezmu@nebrija.es (A.G.); jvinolas@nebrija.es (J.V.)

**Keywords:** Arduino, data logger, low cost, accelerometer, automobile, dynamics, MEMS

## Abstract

This project addresses the need for the implementation of low-cost acquisition technology in the field of vehicle engineering: the design, development, manufacture, and verification of a low-cost Arduino-based data acquisition platform to be used in <80 Hz data acquisition in vehicle dynamics, using low-cost accelerometers. In addition to this, a comparative study is carried out of professional vibration acquisition technologies and low-cost systems, obtaining optimum results for low- and medium-frequency operations with an error of 2.19% on road tests. It is therefore concluded that these technologies are applicable to the automobile industry, thereby allowing the project costs to be reduced and thus facilitating access to this kind of research that requires limited resources.

## 1. Introduction

Currently, one of the most common electronic development platforms is Arduino. Arduino is a platform composed of open hardware and software. People without an advanced knowledge of electronics or programming may use it, thanks to the great amount of information published on the internet. The low cost of Arduino’s hardware is due to the open access of the platform, as well as to the large number of manufacturers. In addition, it has numerous sensors specifically designed to be used with Arduino. Also, it has good reliability and durability [1] even when used in hostile environments such as that of the automobile.

This paper focuses on the development of a low-cost, <80 Hz frequency data acquisition system for automobile dynamics applications. Accelerometers are commonly used in the study of vehicle dynamics. A study is made here of the most frequently used low-cost accelerometers compared to professional accelerometers. Next, the research justification is shown through the study of the state of the art. The results are contrasted with existing works previously published, considering the different fields involved in the present research. 

In [2], a study of the Arduino application as a general acquisition system for research was presented. A general analysis of the application characteristics was performed, as well as a study of the main existing sensors. The principal characteristics were analyzed for low-frequency applications. The application of this general acquisition system was designed for environmental studies.

In the same way, more specific systems have also been implemented for practical applications. In [3], the design and testing of a high-resolution instrument that monitors the physical properties of coastal marine systems was performed. This project focused on the cost minimization and the maximum personalization capabilities of the Arduino data acquisition system. It had a GPS module and a microSD.

One of the most interesting applications of automobile data acquisition systems is the measurement and control of the relevant parameters in the studies of driving behavior [4,5] and the estimation of the characteristic vehicle parameters [6]. The need to use low-cost technologies that foment access to universities and to companies with reduced budgets arises because of the high costs that are traditionally linked to the acquisition systems. 

The emergence of new, low-cost (open access) technologies has led to a series of studies that try to apply these platforms to automobile data acquisition. Reference [5] describes the use of a mobile phone as an acquisition system. The work is focused on assessing the driving maneuvers using the triaxial accelerometer integrated in the phone. The use of a device as popular as today’s smartphones allows a drastic costs reduction but presents a series of drawbacks compared to specialized acquisition systems, such as limitations on the sampling frequency and the difficulty of using external sensors. In [7], the utilization of a low-cost, three-axis accelerometer was studied for an analysis of the driving behavior of public transport operators. An automatic system was obtained which used five statistic parameters to discriminate aggressive and normal driving behaviors. In [8], the development of a low-cost embarked system (Hardware, Firmware and Software) was shown to evaluate the dynamic movement factors that affect the comfort of public transport systems. For this purpose, a low-cost triaxial accelerometer and a GPS module were used. In [9], a low-cost prototype was presented, whose objective was the detection of vehicle comportment as well as the failure detection. This prototype used a low-cost triaxial accelerometer, a GPS module, and the On Board Diagnostics (OBD) bus communication system. A special study focused on the brakes and gear shifting system of the vehicle was made. All of the aforementioned studies mainly deal with driver behavior and passenger comfort during journeys undertaken on road vehicles such as automobiles and buses. They neither study the numerical parameter estimation of the car components nor the use of high acquisition frequencies.

In addition, low-cost systems have been employed in applications unrelated to the automobile industry, for example, the parameter estimation, using data acquired with a low-cost system. In [10], a study of the displacement estimation caused by earthquakes was made, using low-cost triaxial accelerometers. The estimation was carried out using very low input signal frequencies (0.5 to 2 Hz), low force values (+2 G to −2 G), and a low acquisition frequency (25 Hz).

In summary, there are very few examples of using low-cost acquisition technologies with triaxial accelerometers for frequency ranges over 25 Hz.

Therefore, the objective of our research is the design, development, fabrication, installation, and test verification of a low-cost Arduino-based data acquisition platform, which is to be used in <80 Hz data acquisition frequency in vehicular dynamics with the aid of low-cost accelerometers. In particular, a complete, reusable, and easily configurable system is developed. This system must guarantee data acquisition at high frequency (up to 2 kHz) and an easy installation in any vehicle without an aggressive vehicle modification (it must be easily installable and detachable in any vehicle). A study of the uses of low-cost accelerometers in vehicle dynamics applications is also carried out.

The article is divided into the following sections. Section 2 compares the technical features of Arduino versus professional acquisition systems. Section 3 presents the different results obtained in the tests. Section 4 details a discussion of the results and compares the results obtained by professional acquisition systems and those of the proposed low-cost Arduino-based acquisition system (ADAQ). Finally, in Section 5, the final conclusions of the study are presented. The fidelity of the results obtained by the low-cost accelerometer and the Arduino-based acquisition system is demonstrated. 

## 2. Design and Manufacturing of the Arduino Data Acquisition System (ADAQ)

The design of our Arduino-based acquisition system (ADAQ) should have the main feature of being easily programmable and customizable for its use in different applications of vehicle data acquisition at frequencies up to 2 kHz. For this reason, the maximum reduction of the ADAQ system dimensions without losing its characteristics is sought. This permits the easy installation of all components without welds, thereby increasing or decreasing the characteristics of the system, depending on the need and the budget. Its design is detailed below.

### 2.1. Definition of the System Specifications

The Arduino hardware consists of a printed circuit board with a microcontroller (usually Atmel AVR), analog input and output ports, a communication system for serial programming and communication, and a 3.3 and 5 V power supply system. 

Our acquisition system receives the information provided by the sensors in three ways.

• Analog inputs

The connection used for the transmission of the data taken by analog sensors, as, in our case, the accelerometer ADXL335. The voltage of the signals should have a maximum range of 0 to 3.3 V or 5 V depending on the version of Arduino, which does not allow negative voltage values. Those that do not meet these ranges must be conditioned to avoid damaging the printed circuit board (PCB).

• I2C Protocol (Inter-Integrated Circuit)

The communication protocol used to connect a large number of digital sensors using two signal lines: CLK (Serial Clock) and data line (SDA, Serial Data). Sensors connected to the bus (slaves) have an address, given by the manufacturer, which the acquisition system (master) can identify. One of the problems that can be found when using this communication protocol is that two sensors of the same manufacturer have the same identification address, so the master is not able to know which device is transmitting at any moment. This may be solved by using a multiplexer.

• SPI Protocol (Serial Peripheral Interface)

The communication protocol for digital sensors. It has four connections per device: a Serial Clock (SCLK), Master Input Slave Output (MISO), Master Output Slave Input (MOSI), and a chip select pin (SS) which connects or disconnects the device. This protocol is faster than the I2C, since it has two lines for data transmission. Thanks to the select pin (SS), this protocol does not require the slaves to be identified by an address.

In the Arduino data acquisition system, ADAQ, all three methods are used.

There is a great diversity of Arduino boards. Each of them has specific characteristics. Table 1 shows the characteristics of four of the most commonly used models.

### 2.2. Characteristics

Before designing the data acquisition system, the characteristics that are to be satisfied must be known. The ADAQ must have a high acquisition speed and a large number of analog and digital inputs that enable us to instrument a quarter vehicle, as well as an adequate resolution (bits) for the analog inputs. It must also be compact, economical and reliable. Table 2 shows the characteristics of each of the most common Arduino models based on their technical specifications [11,12,13,14].

Our choice was the Arduino Due board because the clock frequency is more than five times higher than that of Arduino Uno and Mega. This allows us to read more sensor data at the same acquisition speed. In addition, Arduino Due ADC (Analog-to-Digital Conversion) samples the signal with a resolution of 12 bits. It is able to differentiate voltage ranges of 0.806 mV. On the other hand, the other models have a resolution of 10 bits, so they are only able to differentiate intervals of 4.88 mV. In addition, Arduino Due allows us to use up to two analog outputs in order to be able to perform control functions.

### 2.3. Components

The design of our acquisition system allows us to add components as needed, so the cost and hardware characteristics can be adapted to each specific application in vehicle data acquisition. The system consists of a shield that is installed on the Arduino Due board. Different modules may be added to expand the characteristics of the system. The design of this shield is original and there are no existing commercial solutions or prototypes that satisfy these characteristics.

Among the modules that extend the acquisition characteristics of the board is a GPS sensor. This allows us to know the geolocation of the vehicle, which is very useful in road tests, where it is necessary to study the behavior at each approximate point of the circuit. The development and implementation of a differential GPS would obtain greater precision in the vehicle location. The system also has a module for microSD cards. This module allows the system to be autonomous, without needing a computer for acquisition. A Real Time Clock (RTC) gives the time values in which the acquisition was made. These date and time values complement the internal clock of the Arduino Due processor. It also has a Bluetooth module that enables interaction with the system from a mobile device. Finally, an LCD screen can be installed in case it is necessary for the user to obtain information about the status of the ADAQ.

**Figure 1:**
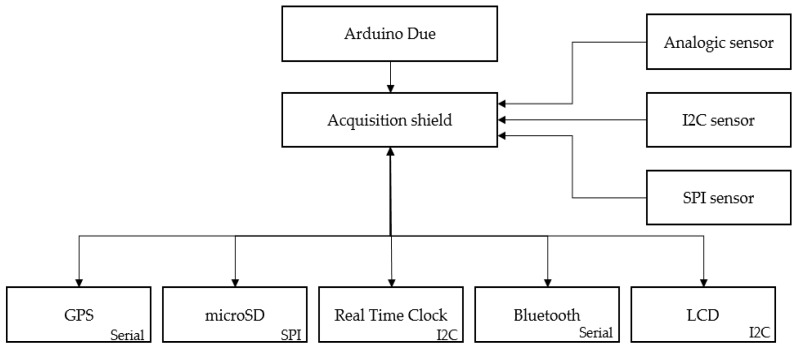
Component communication scheme.

Figure 1 shows an outline of our system’s component communication scheme.

Table 3 shows the necessary and optional components of the system.

The sensors are connected to the shield directly. These can be analog sensors whose output is between 0 and 3.3 V. Other voltage values outside this range must be conditioned so as not to damage the system. Likewise, sensors using the I2C bus can be used. The shield is apt to use 5 V or 3.3 V I2C thanks to the installation of a voltage converter. Finally, the SPI protocol can be used at 3.3 V voltages.

In addition, a box is designed to protect the acquisition system from external factors. This box is manufactured in 3 mm thick methacrylate by laser cutting.

Therefore, the basic system consists of a shield that is connected to an Arduino Due board. With this system, data acquisitions are made. In addition, different modules may be included to augment the characteristics. In this way, the initial outlay is small.

Manufacturing the ADAQ required a budget of 40.12€. The cost of this system increases as modules are added. The unit prices of the components are shown in Table 3. The manufacturing cost of the PCB depends directly on the number of units manufactured. In our case, it was manufactured very cheaply, using a double-sided copper plate.

### 2.4. Software

**Figure 2:**
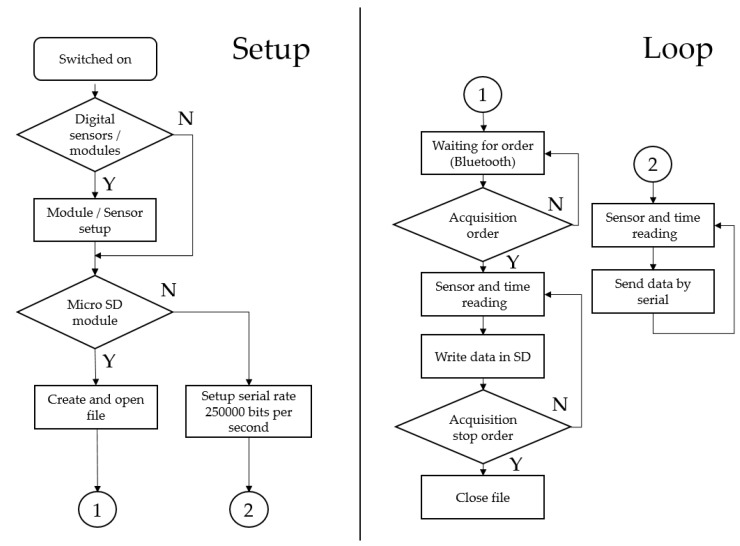
ADAQ software flowchart.

The software developed for the device must be able to be easily modified to suit specific requirements. The code was written using the Arduino IDE. The program flow chart is shown in Figure 2.

After switching on the device, the system begins detecting the installed modules and sensors. Once the setup is completed, the system checks to see if there is a microSD card inserted in the system. If so, a .txt file is created for the acquisition data saved in the future. Otherwise, the serial connection to the computer is established at a rate of 250,000 bits per second. If this software is used, the use of the microSD module requires incorporating the Bluetooth module to be able to manually start and stop the acquisition. In this case, the system waits for a command given by Bluetooth to start sensor reading and SD card writing. When the order (by Bluetooth) is issued to stop the acquisition, the .txt file is closed, leaving all the values saved. If the file is not closed, the data will not be saved. That is why the use of the microSD module involves incorporating a Bluetooth module. On the other hand, if the acquisition is being made by the serial port, the acquisition system will enter into an infinite loop of reading and sending data from the sensors. The computer is used to stop and save the data.

All sensors must be calibrated. The results of the calibration must be applied in data post-processing. The calibration process does not change the sensor output. 

In our future works, microcontroller interruptions will be included to obtain constant and user-defined acquisition speeds.

### 2.5. DAQ Shield

To facilitate the component integration of Table 3, a compact connection interface (Shield) was internally designed and manufactured for its installation on Arduino Due. This facilitates size reduction and the easy integration of the acquisition system in the vehicle without having to be invasive. 

The PCB board design allows an easy installation on the Arduino Due board. The shield is positioned above the Arduino board connecting the pins electrically without soldering. One of the main features of the ADAQ is its ability to receive additional modules that increase the characteristics of the acquisition system. These modules are connected to shield terminals.

**Figure 3:**
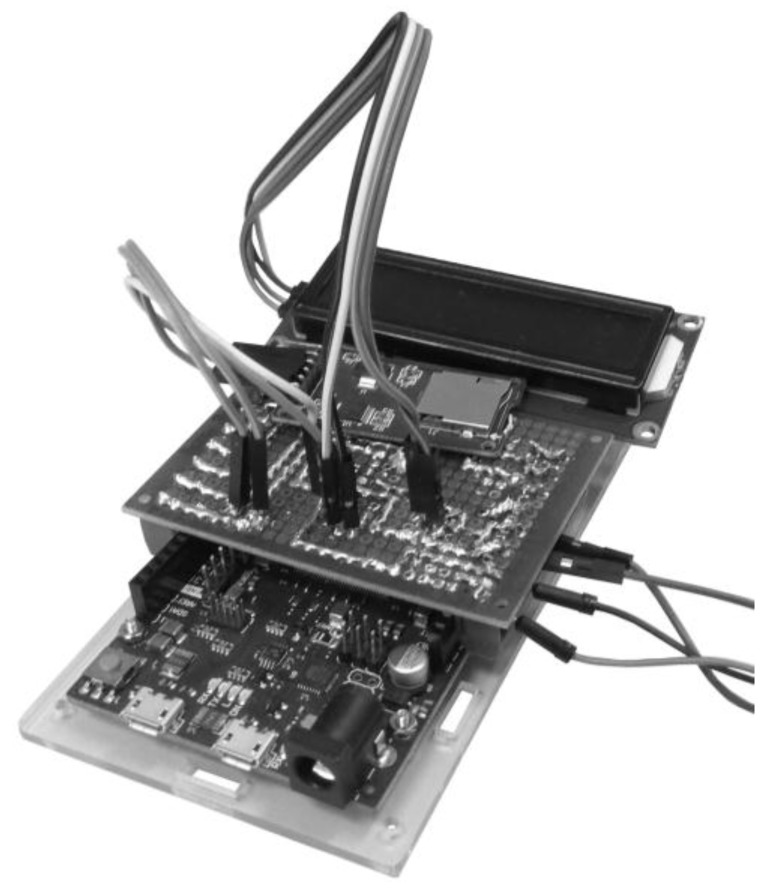
First ADAQ prototype.

**Figure 4:**
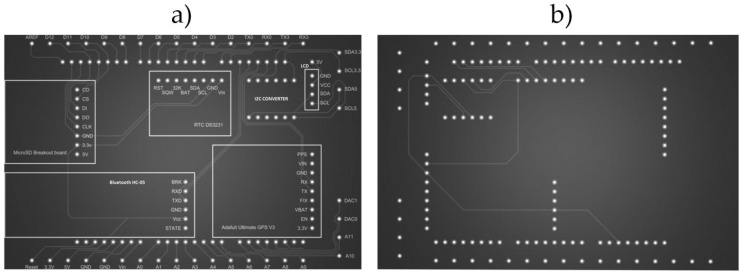
Final ADAQ PCB design. (**a**) Top layer of the PCB; (**b**) Bottom layer of the PCB.

The shield was made in a two-layer PCB with a size of 100 × 70 mm. Figure 3 illustrates the first prototype of the PCB. The output board connectors are connected by screw connection. The system has a logic converter for the I2C protocol. This allows us to be able to use sensors with a 5 V operation voltage when Arduino Due native voltage is of 3.3 V. Figure 4 shows the final PCB design. The board has a top silk screen where the placement of all sensors is shown. The drills within these areas have socket pins to allow an easy connection. Also the pins that connect to the Arduino Due board include pin headers for installation as a shield. Finally, external drills are provided by screw terminals to connect the external sensors comfortably and safely.

The shield has a space for the installation of a GPS (Adafruit Ultimate GPS V3). This module has an update frequency of 1 to 10 Hz. It is also capable of capturing up to 22 satellites with a speed and position error of 0.1 m/s and 1.8 m, respectively. It includes an internal antenna and is a low-cost module that is used to know the approximate position of data acquisition. On the other hand, it has a Real-Time Clock (DS3231) that counts seconds, minutes, hours, date of the month, month, day of the week, and year. It is a low-cost, accurate I2C real-time clock (±2 ppm) with an integrated temperature-compensated crystal oscillator and crystal. It is used in acquisitions made over long periods of time. For storing SD data, the MicroSD Card Breakout Board module is used. This module connects a microSD card with the Arduino board using the SPI protocol. It saves all data in a .txt-formatted document. Finally, the Bluetooth module HC-05 is used, which is one of the most employed and allows us to communicate with the system to start and stop the data acquisition. These modules will be tested in future work with in-vehicle tests.

Table 4 shows the characteristics of the shield.

## 3. Materials and Methods 

In order to know to what extent the ADAQ system can be used in the field of vehicle data acquisition it is necessary to test its performance by comparing it with a professional acquisition system. In our case, the comparison was made with the B&K Photon +, which is a system of acquisition with four channels connected to piezoelectric accelerometers. The acquisition tests were performed with the ADAQ system together with different low-cost accelerometers and the professional acquisition system of B&K, at the same time. In this way, the acquisitions between the two systems were directly compared. To perform the tests, the ADAQ system was used without additional modules, with the exception of the microSD module. This was used only in the first test. The other tests were communicated to a computer via a serial connection. Three models of low-cost MEMS accelerometers were used. The MPU6050, the ADXL345, and the ADXL335. These are the most popular low-cost sensors on the market. The MPU6050 sensor (InvenSense, California, United States) has been used in many applications, such as fall detection systems [15] and measurement balance systems [16]. It contains a triaxial MEMS accelerometer and an MEMS gyroscope. It has a 16-bit digital analog converter allowing high resolution. The ADXL345 sensor (Adafruit Industries, New York, United States) is a small, low-power, three-axis accelerometer with high resolution (13-bit) measurement up to ±16 g. The output data was formatted as 16-bit plug-ins and was accessible via an SPI (three or four-wire) or I2C. The ADXL345 has the ability to measure the static acceleration of gravity for tilt sensing applications, as well as the resulting dynamic acceleration of motion. Finally, the ADXL335 (Adafruit Industries, New York, United States) is an accelerometer with small dimensions, low power and with three axes with voltage-conditioned signal outputs. It measures the acceleration with a minimum scale range of ±3 g. It can measure the static acceleration of gravity in tilt-sensing applications, as well as the dynamic acceleration resulting from motion, shock, or vibration. The data, sent by serial connection from the ADAQ, were saved in .txt files. The time signal was recorded in microseconds and the acceleration signal in ms^−2^ (the acceleration values are modified subtracting the gravity acceleration). To calculate the acquisition frequency, a MATLAB program was used. The code read the number of samples per second of the generated files.

Brüel Photon + is an analyzer for measurement, recording, and post-processing of noise and vibration signals. Which is used in combination with the RT Pro Photon 7.20 software. This allows to configure the sensors as well as perform data acquisition in a simple way, giving the possibility of selecting different ranges of acquisition frequencies between 20 Hz and 192 kHz. In this work, an acquisition frequency of 2560 Hz was used in all tests for the B&K Photon+ System. This setup saved time and acceleration signals in .txt files to be compared to the data from the low-cost sensors. The characteristics of the system are detailed in Table 5.

The main limitation of this system is the number of sensors that can be connected at the same time. It cannot acquire more than four sensors simultaneously. In our case, an accelerometer Type 4534-B model was used. This sensor has a frequency range of 0.2 Hz to 12,800 Hz, low noise, low sensitivity (0.9691 mV/ms^−2^) to environmental factors, and a weight of 8.6 g. 

For performing laboratory tests where low-cost accelerometers were compared to professional Brüel accelerometers (Brüel & Kjaer Ibérica, Madrid, Spain), the Brüel exciter LDS V555 was used with a signal amplifier.

**Figure 5:**
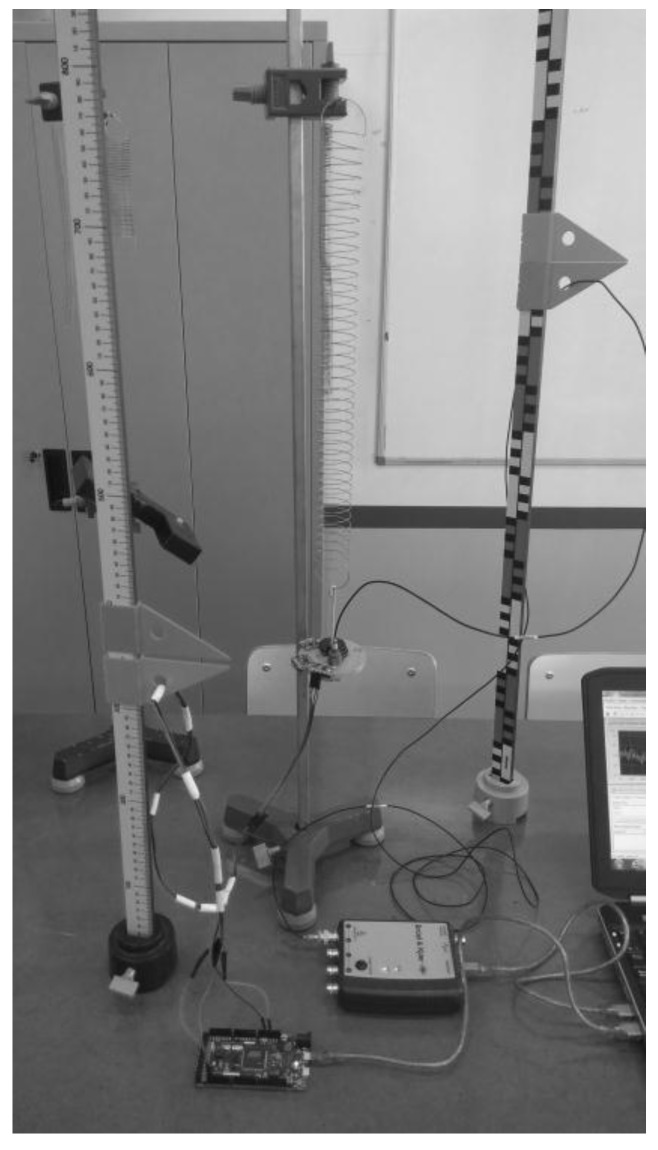
Accelerometers low-frequency tests using springs and masses.

The accelerometers are studied at different frequencies and amplitudes of a sinusoidal wave. This signal is produced by the Photon + equipment, amplified by a LDS PA100E CE device and converted into displacement by the Brüel LDS V555 electromagnetic exciter. Performing the tests with this equipment allowed us to observe the behavior of each accelerometer for different frequencies (10 Hz–150 Hz) and for different amplitudes. In this way, the different limitations of each sensor were known. For tests at frequencies below 10 Hz, we used a mechanical mass spring system to obtain sinusoidal signals. The electromagnetic exciter was not designed to excite these low frequencies. In Figure 5, the test is shown using the mechanical system. 

**Figure 6:**
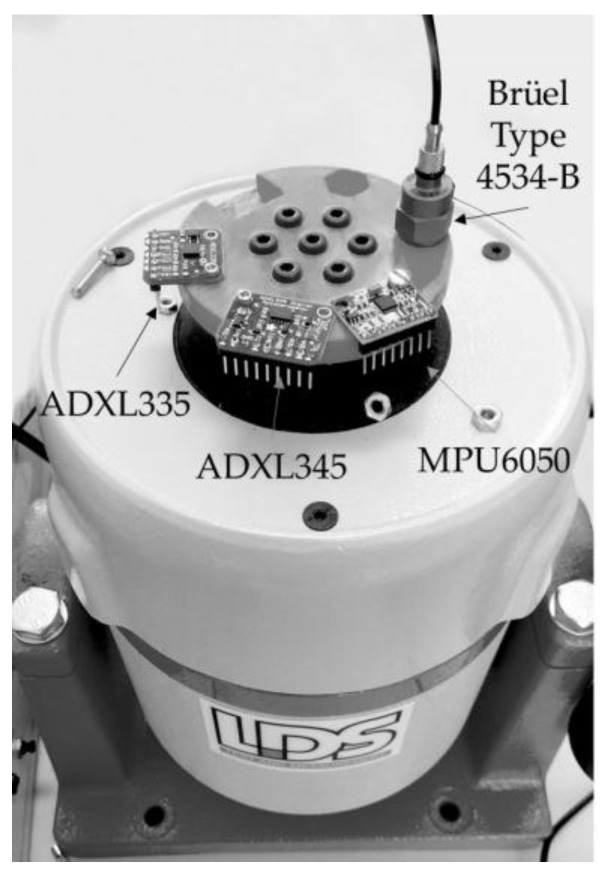
Accelerometers fixed to the electromagnetic exciter.

The accelerometers were assembled with a support screwed into the exciter. Low-cost accelerometers that were incorporated in small PCBs were fixed by screws to the support. Professional accelerometers were fixed by beeswax. This part was screwed to the exciter by means of seven screws. Figure 6 shows the fixation of the accelerometers to the electromagnetic exciter.

To carry out tests on the spring mass system, a characterization of the spring was made in the laboratory. The masses were adjusted to obtain oscillation frequencies of 1 Hz and 2 Hz. The sensors were mounted on a support in the lower end of the spring that was free to oscillate. 

The tests performed with the exciter were as follows. The experimental study of maximum acquisition frequencies for different numbers of analogue and digital sensors was carried out. The study was divided into five tests that are explained below.

Test 1 consisted of the analog signals acquisition through a serial communication with the computer. It began with the acquisition of a single signal, gradually increasing their number. The acquisition speed was recorded in function of the number of the analog sensors acquired simultaneously, so as to be able to know the acquisition speed limitation and to study the behavior of the system.

Test 2 was similar to Test 1 but used the microSD module instead of the serial connection to the computer. Predictably, it could be assumed that the acquisition speed using this method would be inferior to that obtained using the serial port. All the data was registered in a file txt in each interaction. This procedure required additional time that increased the total elapsed time between readings.

In Test 3, a study of the maximum acquisition speed using ADXL345 accelerometers was carried out. These accelerometers have three reading axes. Different numbers of ADXL345 accelerometers were sampled at the same time with different number of purchased axes. In this way, the behavior of the acquisition system speed was studied in function of the number of sensors and axes of each acquired sensor.

Test 4 was carried out comparing the different models of low-cost triaxial accelerometers (ADXL335, ADXL345 and MPU6050) with the professional piezo-electric accelerometer for the most relevant excitation frequencies and wave amplitudes in vehicles [17], using the electromagnetic exciter for frequencies of 10 Hz and 15 Hz and using the spring mass system for the frequencies of 1 Hz and 2 Hz. For the synchronization of both systems, a slight punctual impact on the support of the accelerometers is provided. The system also admits the use of a trigger, but has not been used in these tests.

Test 5 shows a frequency study of the MPU6050. This study included frequencies between 0 and 150 Hz that were produced by the exciter. The excitation frequency was increased every 5 Hz, maintaining constant the amplitude of the electric wave that the B&K Photon + system provides to the amplifier. In this way, the behavior of the sensor could be observed in a wide range of frequencies.

Finally, in Test 6, a vehicle test was made to compare the low-cost accelerometer with better performance (MPU6050) to the professional B&K system. In this way, the behavior of these sensors in a real field was studied. Both accelerometers were placed inside the cab of the car, positioning them in the mass center of the vehicle. A road speed bump test was carried out to check if the MPU-6050 low-cost accelerometer performance, detecting sudden acceleration and frequency, as well as the reliability of the measurement change in comparison to a professional sensor. Test 6 was performed at a speed of 30 km/h, going over one speed bump. The vertical acceleration was measured to compare the operation of both sensors. The longitudinal acceleration was also acquired to record the driving events.

To compare the operation of the accelerometers, the Root-Mean-Square Error (RMSE) was used. The RMSE is a measure that is usually used to compare two values, a reference value and a measured value [18,19]. In our case, the reference was the acceleration acquired by the professional system and the measured value was the acceleration sampled with the ADAQ and with different low-cost accelerometers. The RMSE represents the sample standard deviation of the differences between both signals. This is a measure of precision that allows comparing errors between several samples for particular data and not between datasets. The units of the RMSE are ms^−2^.

(1) $$\mathrm{RMSE}=\sqrt{\frac{1}{N}\overset{N}{\underset{i=1}{\sum }}{\left(O_{i}-f_{i}\right)}^{2}}.$$

Equation (1) shows the mathematical formula where *O_i_* is the acceleration taken by the B&K system, *f_i_* is the acceleration sampled by the low-cost sensors, and *N* is the number of samples. Since the two acquisition systems take data at different frequencies, a data interpolation was performed. In this way, two acceleration values were obtained for the same time value. 

The normalization of the RMSE facilitated the comparison between datasets with different scales and provided a more representative value of the functioning of each sensor. Normalized RMSE (NRMSE) is usually expressed as a percentage, where the lower values indicate a lower residual variance. Equation (2) shows the mathematical formula of the NRMSE, where *y_max_* is the maximum value sampled and *y_min_* the sampled minimum value.

(2) $$\mathrm{NRMSE}=\frac{\mathrm{RMSE}}{y_{max}-y_{min}}$$

## 4. Results

The results obtained in the study are shown below.

**Figure 7:**
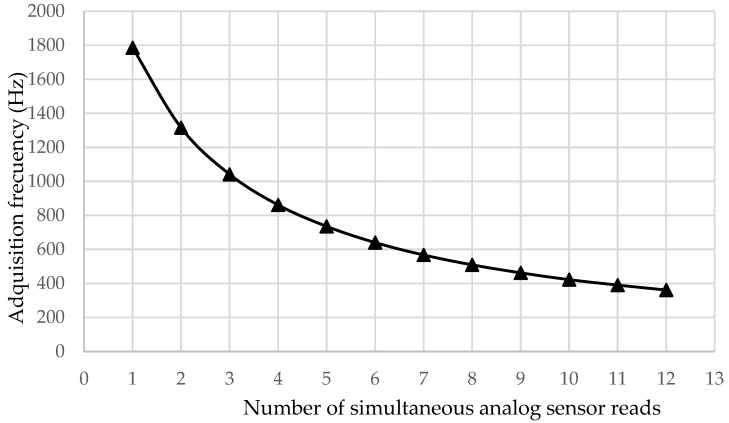
Frequency dependence of analog pins with a serial connection.

Figure 7 shows the obtained values in Test 1, in which different numbers of analog inputs were acquired simultaneously. The evolution of the acquisition speed depending on the number of inputs was observed. The behavior of the system was not linear. The influence on the acquisition speed was higher when fewer sensors were connected. When there was a greater number of connected sensors, the variation after incorporating an additional one was smaller.

**Figure 8:**
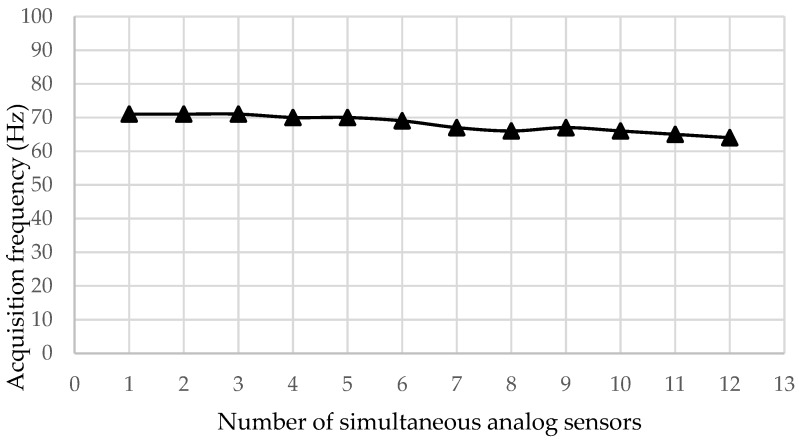
Frequency dependence of analog pins with the SD module.

Figure 8 shows Test 2, a similar experiment to Test 1, but using the microSD module to store the obtained data on a microSD. An external computer was not required to perform the acquisition. In this case, it can be seen that the acquisition speed remained practically constant. Different numbers of sensors were acquired with the security of obtaining an almost constant acquisition speed. This was because it was the SD module which limited the acquisition speed and not the Arduino board microcontroller.

**Figure 9:**
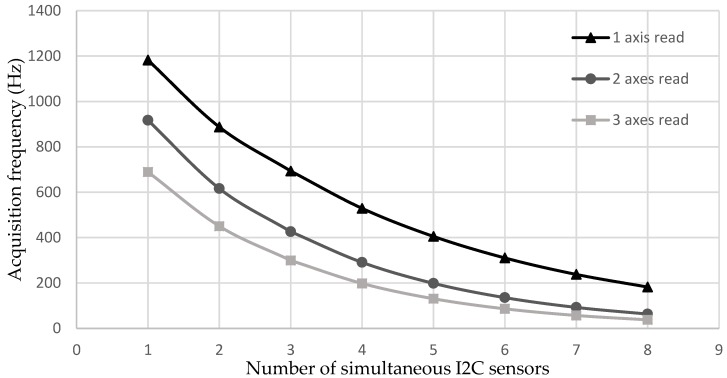
I2C accelerometer.

Figure 9 shows Test 3, where ADXL345 accelerometers were connected by the I2C protocol. Different numbers of accelerometers were acquired at the same time and different numbers of sampled axes (1 to 3) were observed. These were the *x*, *y*, and *z* axes of triaxial accelerometers. Depending on these values, three curves were obtained. The behavior was similar to that of Figure 7. When more axes were acquired at the same time, a lower acquisition frequency was obtained. At the same time, with a greater number of sensors, the acquisition frequency was also reduced.

The results from the low-cost accelerometers ADXL335, ADXL345, and MPU6050 tests are presented below, on Test 4.

**Figure 10:**
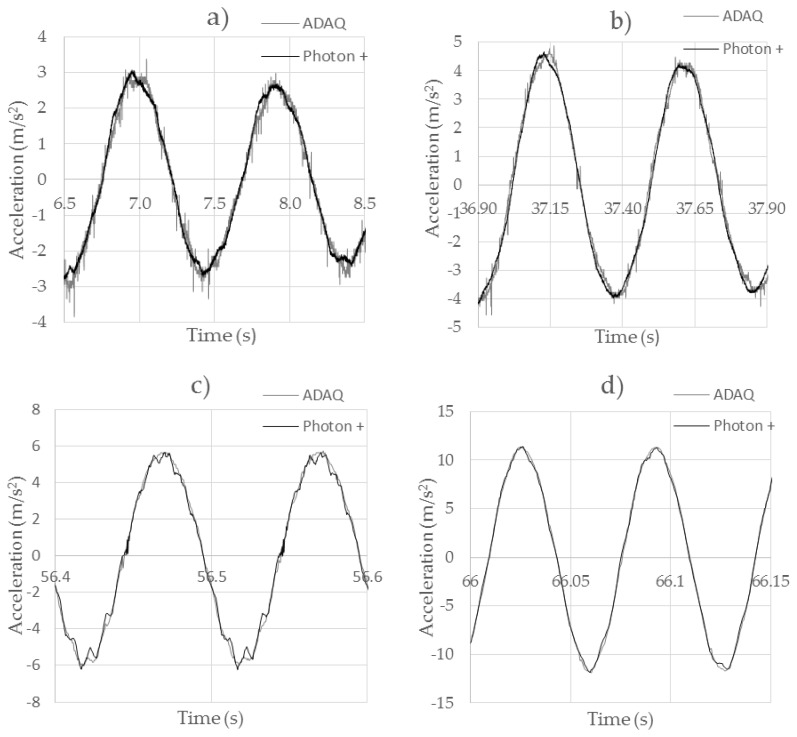
Comparison between the ADXL335 and Brüel Type 4534-B accelerometers. (**a**) 1 Hz test; (**b**) 2 Hz test; (**c**) 10 Hz test; (**d**) 15 Hz test.

Figure 10 shows the behavior of the ADXL335 analog accelerometer for different excitation frequencies that varied between 1 Hz, 2 Hz, 10 Hz, and 15 Hz. When the accelerometer was excited at frequencies of 1 Hz (Figure 10a), two differences could be observed compared to the professional accelerometer. On the one hand, the values obtained by the low-cost accelerometer had a noise level of up to ±6 m/s that was not present in the original signal. This noise caused maximum acceleration peaks that did not correspond to the reality. This error could be corrected through the application of a signal processing, using filters. On the other hand, it was observed that in the acceleration peaks the behaviors between both accelerometers were slightly different. Thus, this accelerometer could be discarded for high-accuracy applications. If the excitation frequency was increased to 2 Hz (Figure 10b), the noise level of the accelerometer started to decrease, as did the discrepancies in the high acceleration zones. In Figure 10c, it was observed that the electromagnetic exciter was not able to provide us with a clean sinusoidal signal. This helped us to appreciate that the low-cost accelerometer was not capable of accurately registering an abrupt change in acceleration with the same precision as the Brüel accelerometer. In Figure 10d, the behavior of both accelerometers can be observed when they are excited at a frequency of 15 Hz and an amplitude of acceleration >1 G. In this case, a better behavior was observed, without the existence of an appreciable noise or any type of lag between both signals.

**Figure 11:**
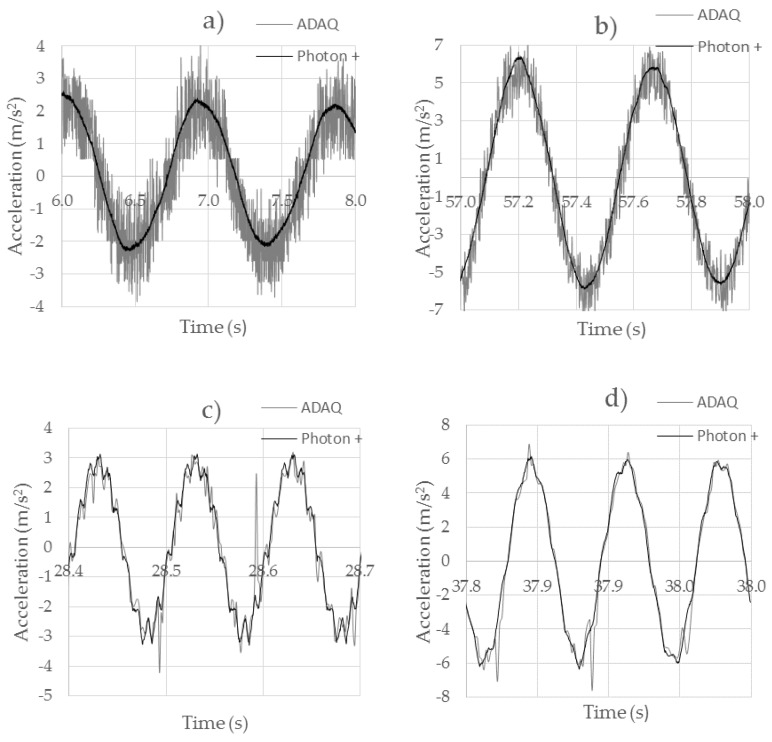
Comparison between the ADXL345 and Brüel Type 4534-B accelerometers. (**a**) 1 Hz test; (**b**) 2 Hz test; (**c**) 10 Hz test; (**d**) 15 Hz test.

Figure 11 shows the behavior of the ADXL345 accelerometer shown at different excitation frequencies that vary between 1 Hz, 2 Hz, 10 Hz and 15 Hz. For excitation frequencies of 1 Hz (Figure 11a), a signal similar to that acquired with Brüel was observed, but with an unacceptable signal noise level, reaching up to ±1.5 m/s^2^ for accelerations of 2.3 m/s^2^. For periodic sinusoidal signals, it could be corrected by applying filters. For accelerations of 5 Hz (Figure 11b), there was a decrease in the noise level to approximately ±1 m/s^2^. No noticeable phase shifts were observed with the original wave. When it was excited at 10 Hz frequency (Figure 11c), the exciter was no longer able to perform a clean excitation. We could appreciate the disappearance of noise that was present for low excitation frequencies, as well as a better behavior in response to abrupt acceleration variations, showing that the ADXL345 had a higher fidelity than the ADXL335 accelerometer. In spite of this, there were deviations of up to 0.2 m/s^2^ for signals of ±3 m/s^2^. The point values were completely erratic, because of a low-cost sensor malfunction. For 15 Hz excitations (Figure 11d), a behavior similar to the previous one was obtained, with an imprecise behavior at specific moments, but without the existence of noise.

**Figure 12:**
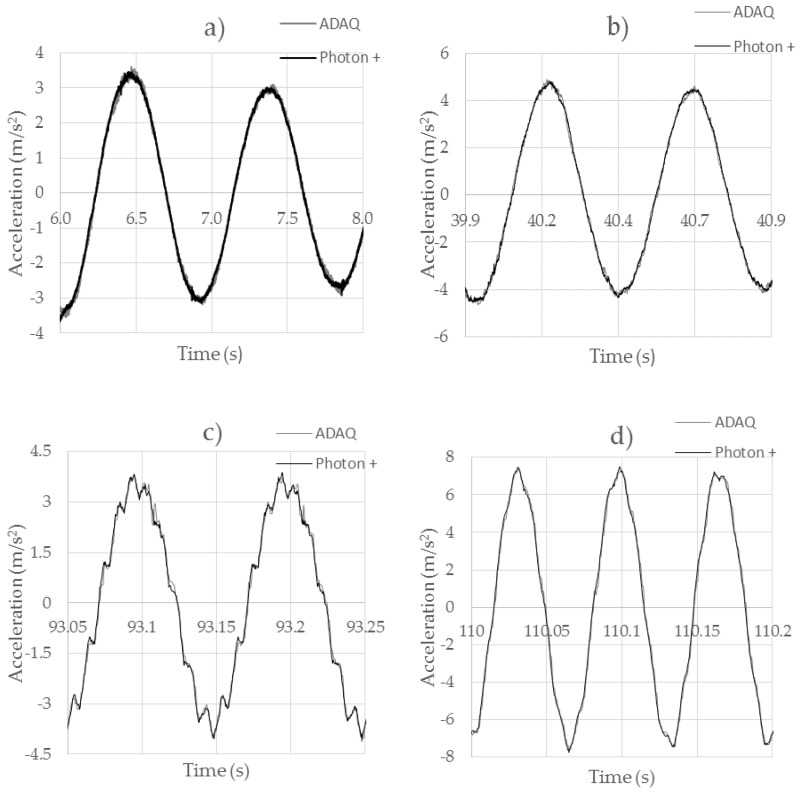
Comparison between the MPU6050 and Brüel Type 4534-B accelerometers. (**a**) 1 Hz test; (**b**) 2 Hz test; (**c**) 10 Hz test; (**d**) 15 Hz test.

Figure 12 shows the behavior of the MPU6050 accelerometer for excitation frequencies of 1 Hz, 2 Hz, 10 Hz, and 15 Hz. For excitation frequencies of 1 Hz (Figure 12a), very accurate results were obtained, comparable to those obtained with the Brüel professional equipment. The signal was clean, without the presence of noise. There was no lag between the two signals. When the frequency was increased to 2 Hz (Figure 12b), very accurate and noise-free results were obtained in the same way. When it was excited with a frequency of 10 Hz (Figure 12c) and the exciter was not able to give a clean sinusoidal signal, we still had a very high accuracy even with the abrupt changes of acceleration. At 15 Hz frequencies (Figure 12d), an excellent behavior was observed, being practically identical to that obtained with the professional accelerometer. The sensor had a good resolution and an excellent performance.

Table 6 shows the NRMSE values of Test 4. For each low-cost accelerometer, errors were calculated for each frequency. It can be observed that the results of the MPU6050 sensor are closer to the ones of B&K, with a NRMSE between 1.49% and 1.85%. On the other hand, the ADXL345 had major errors of up to 8.47%. The decrease in its error as the acquisition frequency increased was due to the appearance of noise at low frequencies. This noise became smaller as the frequency increased. Finally, the ADXL335 s located between both, having a good behavior with an NRMSE that was between 2.31% and 2.92%. 

**Figure 13:**
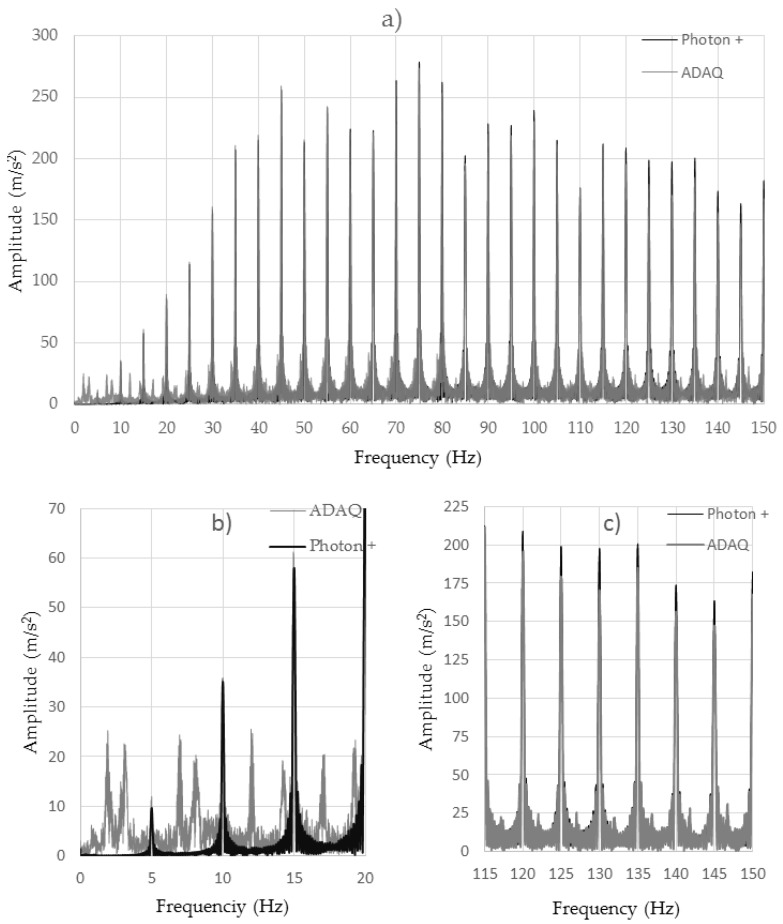
Frequency analysis of a sinusoidal signal that varies from 0 to 150 Hz every 5 Hz acquired by the accelerometer MPU6050. (**a**) Complete frequency range; (**b**) Low frequency range detail; (**c**) High frequency range detail

For Test 5, Figure 13a shows a frequency-dependent analysis for the MPU6050 accelerometer with the Brüel driver. Frequencies ranging from 0 to 150 Hz varied every 5 Hz. A coincidence between the two samples was seen. The existence of erratic excited frequencies was observed in the Arduino accelerometers for low frequencies (Figure 13b). This was due to the existence of vibration modes typical of the manufacture of these low-cost accelerometers which are installed on a small PCB board. The rest of the excited frequencies coincided with those of the professional piezoelectric accelerometer. On the other hand, there was an observed loss of amplitude in the signal acquired by the ADAQ with low-cost accelerometers as compared to that obtained with the professional accelerometer Brüel Photon + (Figure 13c). This loss of amplitude corresponded to the peaks of the signal that were lost because of the lack of frequency acquisition of the ADAQ. For periodic signals, this error can be easily solved by approximating the signal to a sinusoidal wave. This cannot be done for non-periodic signals

**Figure 14:**
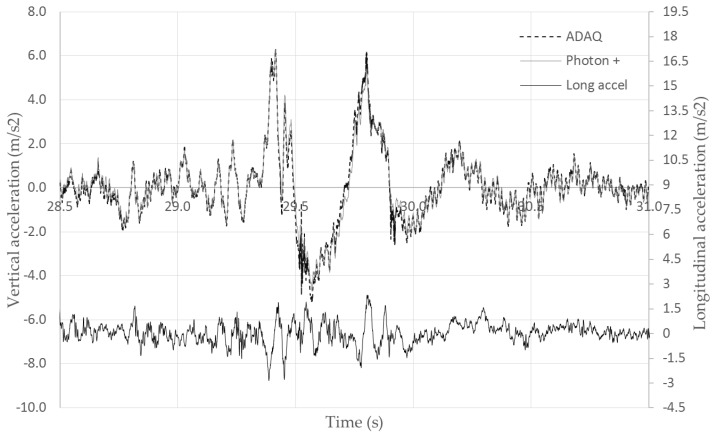
Test 6 acquisition: vehicle passing over one test bump.

For Test 6, Figure 14 shows the excellent performance of the low-cost MPU6050 accelerometer compared to the professional one. The NRMSE error was 2.19%, which was bigger than the maximum of 1.85% present in laboratory tests. This was due to the presence of a large bandwidth of the excitation frequencies in road tests compared to laboratory conditions, as it can be notice in Figure 14. The MPU6050 accelerometer was able to sample the irregularities of the road (corresponding to the highest frequencies of the signal) as well as the low frequencies (caused by the road speed bump) very precisely.

## 5. Discussion

The data acquisition system based on the Arduino platform (ADAQ) allows its use in vehicular dynamics applications. Its data acquisition frequency can reach up to 2 kHz with a single sampled sensor. It has a good resolution and ability to communicate via the SPI and I2C communications buses.

According to the tests performed, the system shows an inverse relationship between the acquisition speed and the number of inputs. Therefore, if it is necessary to acquire a large number of sensor signals, and more than one ADAQ system must be used at the same time. In this case, a system of synchronization between all the systems must be operated. On the other hand, using a computer for sampling rates greater than 70 Hz is recommended, because the use of an SD card data storage slows down the acquisition system.

The tests performed on the low-cost accelerometers show a great performance when compared to the professional piezoelectric accelerometers. The MPU6050 excels the others with a maximum NRMSE of 1.85% in laboratory tests and 2.19% in road tests. It can be used to sample accelerations in <80 Hz frequency applications with a perfect operation up to 80 Hz. Differences exist with respect to professional systems for frequencies higher than 80 Hz, where the peaks in the sinusoidal signals begin to be lost because of the limitation of the acquisition speed.

The advantages of the ADAQ over acquisition systems based on mobile technology is its ability to customize to specific projects. In the case of automotive dynamics, ADAQ allows data acquisition with several accelerometers installed in different parts of the vehicle at the same time. The acquisition system based on the mobile phone does not allow the connection of external sensors. The ADAQ allows acquisition speeds of up to 2 kHz for some specific applications, higher than the mobile phones acquisition frequencies of 100 Hz. In addition, ADAQ is a specific acquisition platform that enables its installation on the vehicle during the period of time that the investigation lasts without having to mount and dismantle, which is something important when you have a considerable number of connected sensors. 

**Figure 15:**
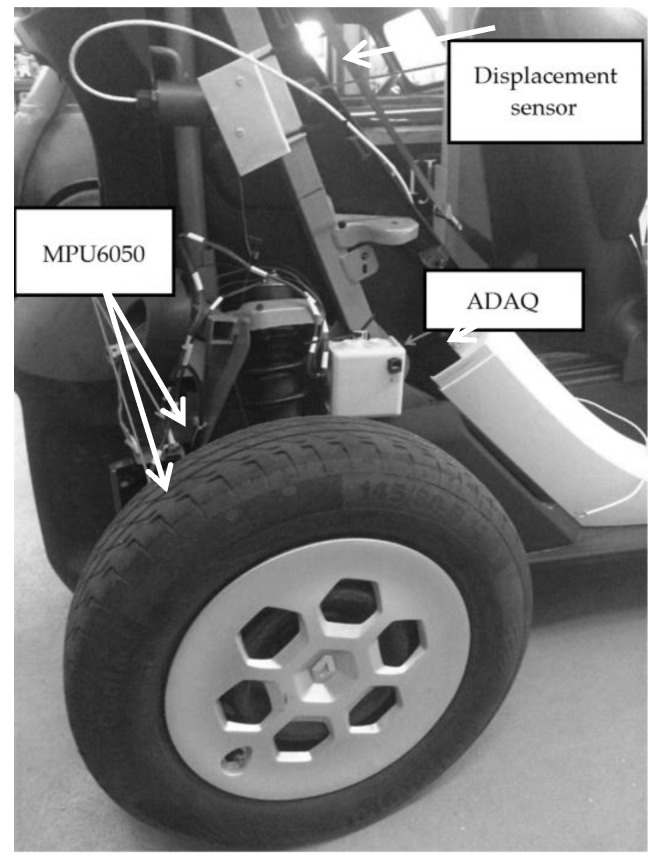
Sensors and ADAQ installed over Renault Twizy.

In future work, on-board tests will be carried out to study the suspension dynamics of a Renault Twizy using the ADAQ system together with MPU6050 accelerometers and displacement wire sensors, as shown in Figure 15. The accelerometers shall be installed in the sprung and unsprung mass of the vehicle. The acquisitions will be made on a straight road with a road bump, sampling accelerations and displacements. A model will be made for the estimation of the displacements through the accelerations taken as in [10,20,21]. The results obtained will be compared to the values acquired by the wire and low-cost ultrasonic sensors [22]. The incorporation of the OBDII communication between the ADAQ and the vehicle will be studied [23,24]. Finally, once the model is verified, an estimation of the parameters of the stiffness of spring K and the coefficient of damping C [25] will be made. On the other hand, the ADAQ system will be used together with MPU6050 accelerometers to conduct a road study by dynamic analysis. For these tests, we propose to use a GPS to differentiate the road sections [26].

## 6. Conclusions

The main objective of this work was to design and build an Arduino data acquisition system (ADAQ) to be used in vehicular dynamics applications. Such a system allows to carry out low-budget research, since it is not necessary to buy expensive equipment and instrumentation sensors. Thus, a researcher who does not have the benefit of sufficient resources may carry out experimental tests. Particular attention was paid to achieving a modular system that would allow us to increase the specifications of our system according to our needs.

It is concluded that the low-cost ADAQ is a valid system for data acquisition in dynamic automotive applications that enables acquisition frequencies up to 2000 Hz, with a 12-bit resolution and an input voltage between 0 and 3.3 V, in addition to sensors with I2C and SPI protocols. Its principal virtues are its low cost and great portability. There are some system limitations for high frequencies when one seeks to acquire a large number of signals at the same time. In such cases, the use of several ADAQs will be used. Low-cost accelerometers (specifically MPU6050) have an excellent performance up to 80 Hz, achieving extremely accurate results with a maximum NRMSE of 1.85% in laboratory tests and 2.19% in road tests compared to professional piezoelectric accelerometers. For higher frequencies, there are losses in some of the signal peaks because of the limitation in the acquisition speed. 

## Figures and Tables

**Table 1:** Technical characteristics of Arduino UNO, Nano, Mega and Due.

Characteristics	Arduino UNO	Arduino Nano	Arduino Mega	Arduino Due
Microcontroller	ATmega328	ATmega328	ATmega1280	AT91SAM3X8E
Clock frequency	16 MHz	16 MHz	16 MHz	84 MHz
Analog inputs	8	8	16	12
Analog output	0	0	0	2
Digital input/output	22	22	54	54
Analog signals range	0 to 5 V	0 to 5 V	0 to 5 V	0 to 3.3 V
Analog signals resolution	10 bit/4.88 mV	10 bit/4.88 mV	10 bit/4.88 mV	12 bit/0.806 mV

**Table 2:** Features of different Arduino models.

Characteristic	Arduino UNO	Arduino Nano	Arduino Mega	Arduino Due
Compact	xx	xxx	x	x
Low cost	xx	xxx	xx	x
High acquisition frequency	x	x	x	xx
High resolution	x	x	x	xxx
Many digital inputs and outputs	x	x	xx	xx
Many analog inputs	x	x	xxx	xx
Analog outputs	-	-	-	x

**Table 3:** Arduino Data Acquisition System (ADAQ) Components, function and prices.

Component	Nature	Function	Commercial	Price (€)
Arduino Due	Obligatory	ADAQ	YES	15.12
DAQ Shield	Obligatory	ADAQ	NO	15
DAQ box	Obligatory	ADAQ	NO	10
GPS module	Optional	Location	YES	51
MicroSD module	Optional	Data saving	YES	7.5
RTC module	Optional	Time recording	YES	1.1
Bluetooth module	Optional	Control	YES	3.06
LCD module	Optional	Control	YES	3.13
ADXL335	Optional	Sensor	YES	3.36
ADXL345	Optional	Sensor	YES	1.08
MPU6050	Optional	Sensor	YES	1.47

**Table 4:** ADAQ characteristics.

Arduino Data Acquisition Shield	
Analog inputs	12 (0 to 3.3 V)
Analog outputs	2 (0 to 3.3 V)
digital i/o	15
Alimentation	5 V, 3.3 V and GND
I2C	5 V and 3.3 V
Analogic reference	Yes
Reset	Yes

**Table 5:** Brüel Photon + characteristics.

Characteristics	Brüel Photon+
Analog inputs	4
Analog output	1
Analog signals range	±10 V
Analog signals resolution	24 bit

**Table 6:** NRMSE of low-cost accelerometers together with the ADAQ versus the B&K system.

Accelerometer	1 Hz	2 Hz	10 Hz	15 Hz
**ADXL335**	2.92%	2.51%	2.21%	2.13%
**ADXL345**	8.47%	5.38%	6.30%	4.51%
**MPU6050**	1.49%	1.49%	1.85%	1.67%
